# Mapping of public health courses and training programs in Brazil

**DOI:** 10.11606/s1518-8787.2023057005205

**Published:** 2023-07-18

**Authors:** Frederico Peres, Maria Pasionaria Blanco Centurión, Juliana Monteiro Bastos da Silva, Ana Laura Brandão

**Affiliations:** I Fundação Oswaldo Cruz Escola Nacional de Saúde Pública Sergio Arouca Centro de Estudos da Saúde do Trabalhador e Ecologia Humana Rio de Janeiro RJ Brasil Fundação Oswaldo Cruz. Escola Nacional de Saúde Pública Sergio Arouca. Centro de Estudos da Saúde do Trabalhador e Ecologia Humana. Rio de Janeiro, RJ, Brasil; II Fundação Oswaldo Cruz Instituto Nacional de Controle de Qualidade em Saúde Rio de Janeiro RJ Brasil Fundação Oswaldo Cruz. Instituto Nacional de Controle de Qualidade em Saúde. Rio de Janeiro, RJ, Brasil; III Fundação Oswaldo Cruz Escola Nacional de Saúde Pública Sergio Arouca Rio de Janeiro RJ Brasil Fundação Oswaldo Cruz. Escola Nacional de Saúde Pública Sergio Arouca. Rio de Janeiro, RJ, Brasil

**Keywords:** Health Human Resource Training, Education, Public Health Professional, Public Health, Brazil

## Abstract

**OBJECTIVE:**

To map public health courses and training programs in Brazil and identify, in their curricula and study plans, the level of linkage between the skills and competencies developed by them and the essential public health functions.

**METHODS:**

Descriptive, exploratory study based on collection and analysis of information available on the websites of educational institutions that offer public health courses and training programs in Brazil. Data was collected within the scope of the *I Mapeo de Cursos y Programas de Formación en Salud Pública de América Latina*.

**RESULTS:**

A total of 1,222 public health courses and training programs offered in the country were identified, with unequal territorial distribution but taking place in all federation units. Results revealed a set of challenges to public health professionals’ training, including lack of linkage both between training offer and demand in public health (unequal distribution of courses, concentration of training capacity in capitals and in certain regions, among others), and between theories and training practices (low levels of connection of developed skills and competencies with the essential public health functions, predominance of disciplinary method guided by professional competence centers, among others).

**CONCLUSIONS:**

Overcoming these challenges requires structural, political, and technological efforts, narrowing the gaps between public health training programs’ availability and the demands of health services and programs in the country.

## INTRODUCTION

Public health training is a strategic action for strengthening health systems and, particularly, for the professional development of human resources responsible for the planning, execution, and evaluation of programs, services, and health policies^1,2^. It involves different educational processes, through which one seeks to develop skills and competencies among health professionals/workers, at different levels and with different purposes, articulating academic knowledge with the practice experience, in an interdisciplinary and interprofessional perspective^2^.

Brazil, due to its marked sociocultural heterogeneity, dynamism, and historical inequality, is strategically important for thinking about and understanding the different dimensions and domains related to public health education^5^. This importance is especially evidenced when we consider the systematic incorporation, in recent decades, of new ways of thinking and acting on the health-disease-care processes, in which the social determinants of health play a central role as elements of analysis^3,4,6,7^.

Although Brazilian public health knowledge has advanced significantly in the last four decades, its incorporation into the set of courses and training programs available for the country’s health workforce is still modest^4,5^. This results in a mismatch between the demands of users of health services and programs and the scope of public health courses/training programs, unevenly distributed across different regions of the country^5,8^.

Peñaranda-Correa et al.^9^, when analyzing public health training initiatives in Brazil and other Latin American countries, point out the lack of coherence between theory and practice, especially regarding the limited connections between pedagogical practices and the required competencies to attend demands of professionals and users of regional health systems^9^. As a strategy to overcome this limited connection, the World Health Organization (WHO) coordinated, in the late 1990s, a working group aimed at identifying a set of functions, in the field of public health, considered essential to guarantee that, regardless of the stage of development of a country or region, health systems continue to respond to emerging and priority needs^10^. In this process, nine essential public health functions (EPHFs) were identified which, therefore, should guide strategies for the health workforce development. In 2020, the Pan American Health Organization (PAHO) revised, updated, and expanded the EPHFs, which became eleven^11^.

That being said, and considering the challenges of developing skills and interprofessional competencies among public health profesionals^1,2,6,7,9^, this study aims to map training public health courses/training programs offered in Brazil and identify, in their curricula and study plans, the level of connection between the skills and competences developed by them and the FESPs^10,11^.

## METHODS

This study was based on the results of the *I Mapeo de Cursos y Programas de Formación en Salud Pública de América Latina*^12^, also coodinated by the authors of this article and whose data were collected between November 2021 and April 2022. It was developed through a systematic review of information on public health courses and training programs offered in Latin America. For the present paper, only data referring to Brazilian courses/training programs were analyzed, which represented 53% of all courses and training programs identified in the set of Latin American countries^12^.

Collection of data on the courses and training programs was made on Google Search, in the Portuguese language, initially using the keywords “curso” [course], “programa” [program], “treinamento” [training], “formação” [qualification], and “educação” [education], always associated with the descriptors “saúde pública” [public health] or “saúde coletiva” [collective health]. Afterwards, the same keywords were associated with the descriptor “saúde” [health] with subsequent application of exclusion criteria. Finally, the descriptors “universidade” [university] or “escola de saúde” [school of health] were used associated with the descriptors “saúde pública,” “saúde coletiva,” and “saúde,” for a more comprehensive search on the educational institutions’ websites. All identified courses were included in a Microsoft Excel spreadsheet to check for duplicate entries and subsequent application of exclusion criteria.

The following exclusion criteria was applied to the mapping: a) courses and training programs in medical specialties, dedicated exclusively to clinical practice (even with an interdisciplinary approach); b) courses related to specialties of specific health careers; c) undergraduate and technical training courses; d) self-instructional courses, and e) courses and training programs that did not have information available about the curriculum or study plan.

In the case of technical training and undergraduate courses in public/collective health, exclusion was due to the fact that the study originated from a regional mapping, covering all of Latin America, and not just Brazil^12^. These undergraduate courses represent a reality exclusive to Brazil, which limits their understanding and articulation in the regional context. Technical training courses were not considered in the sample of this study due to the different conceptions that technical training has in Latin America – there are countries where they are medium-level courses, others where they are higher-education courses, with different workloads and duration –, which also makes a regional analysis difficult.

For each course identified, the following information was collected:

- Place: state and municipality where the training institution is located and/or where the course/training program is offered (for courses and programs with face-to-face activities);
- Course/program name;
- Institution name;
- Institution characteristics (public or private);
- Training level: continuing education courses and programs, specialization, residency, masters’ and doctoral degrees;
- Modality: Distance Learning (DL) (structured courses based on distance education resources, with synchronous and/or asynchronous moments and the possibility of including face-to-face meetings), face-to-face, blended (face-to-face, with distance activities, synchronous or asynchronous), or remote learning (100 % remote and synchronous activities);
- Accreditation: if the course has any type of accreditation and which institution accredits it;
- Year of first offer;
- Regularity: regular (annual or semi-annual since the first offer) or occasional (no defined frequency);
- and contact details (website, e-mail, telephone).

Subsequently, the curricula and study plans of each course/program were analyzed using the descriptive content analysis technique^13^, according to education level, identifying in their topics the competencies developed on in each plan or curriculum, as well as the pedagogical strategies used (e.g., lectures, activities mediated by tutors, asynchronous activities with or without mediation, etc.), duly recorded with the aim of describing the courses/training programs. At this stage, the analysis was restricted to courses in the three thematic areas with the highest number of offers. For categorization and systematization purposes, those competencies associated with the EPHFs^10,11^ were considered fundamental, and all others considered complementary.

All (secondary) data collected and analyzed in the study were available, without restrictions, on the Internet, reason why the analysis by an Institutional Review Board (IRB) was not necessary.

## RESULTS

**Figure 1:**
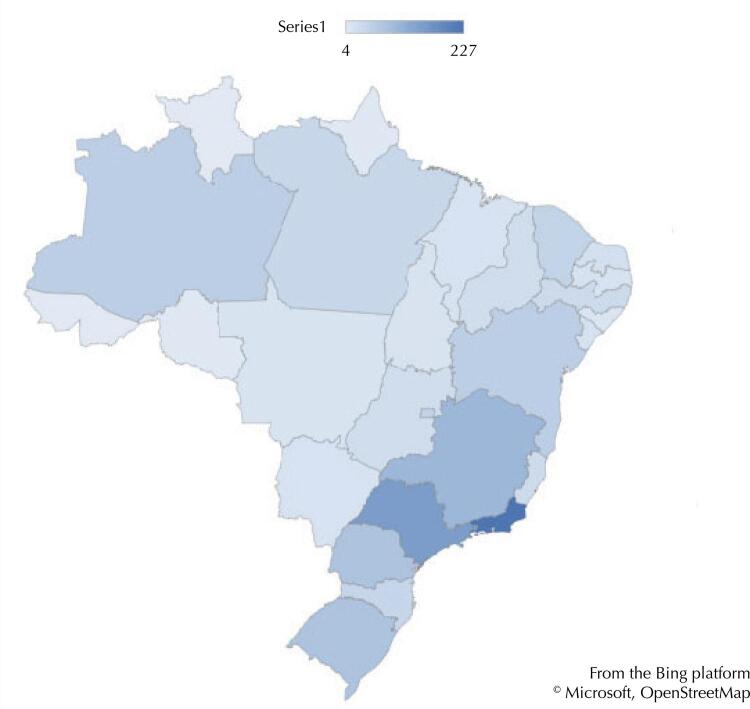
Distribution of public health training courses/programs per federation unit. Brazil, 2022 (n = 1,222).

Data collection identified 1,222 public health courses and training programs offered (or enabled to be offered) in all Brazilian federation units (FU). Although there is a great diversity of courses and training programs, which are offered in all FUs, they run mainly in capitals and in certain states with more resources and installed capacities (Figure 1), such as the states of Rio de Janeiro and São Paulo, which presented the highest number of courses/programs identified.

However, the number of courses offered in the state of Amazonas (n = 56), Northern Brazil, which, despite having a lower installed capacity compared to states in the South, Southeast, and Midwest regions, presented a significant number of courses/programs and, together with the state of Pará (n = 42), has the potential to meet training demands in the Amazon region. This also includes other countries, such as Bolivia, Peru, Colombia, and Venezuela, especially considering that most of the border departments (territorial divisions) of these countries do not offer public health courses or training programs^12^ and that the Amazon has a strategic role in guaranteeing the socio-environmental sustainability of the whole region.

**Figure 2:**
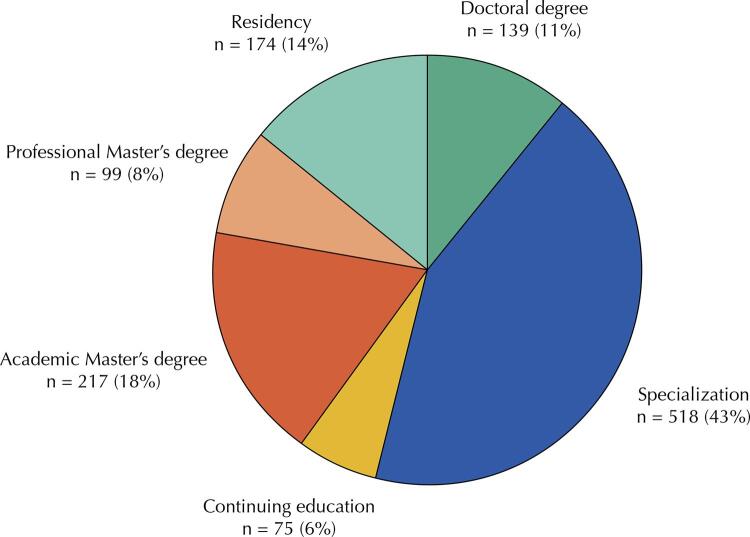
Levels of public health courses/training programs. Brazil, 2022 (n = 1,222).

As for the education level, there was a greater number of specialization courses (n = 518), corresponding to 43% of identified courses. Next, there are the academic master’s courses (n = 217; 18%) and residency (graduate medical or multidisciplinary education) programs (n = 174; 14%) (Figure 2).

It is important to note that 65% of specialization courses identified were offered by private institutions (n = 340). In master’s (academic and professional) and doctoral courses and in residency (medical and multidisciplinary) programs, the logic is reversed, with a prevalence of offers by public institutions (89% academic master’s degree courses; 85% professional master’s degree courses; 88% doctoral degree programs; and 83% residency programs).

With regard to regularity, and considering only the courses and programs that provided this information (n = 965, or 79% of the total offered), 90% of the training programs were regular (annual or biannual periodicity), and the two oldest courses were also regular (annual), whose first offers date back to the year 1970 (master’s and doctoral degrees in Public Health from the Faculdade de Saúde Pública da Universidade de São Paulo – FSP-USP). Approximately 81% of courses and training programs were created (or had their first offer) from 2002 onwards.

Regarding accreditation or the existence of any accreditation system for the courses and training programs identified in the study, none of the institutional websites provided such information, with the exception of the *Curso de Residência Multiprofissional em Saúde Materno-Infantil* [Multiprofessional Residency in Maternal and Child Health Course] of Hospital de Clínicas de Passo Fundo/RS (information on accreditation and re-accreditation). Only 23% (n = 105) of stricto sensu graduate programs (masters’ and doctoral degrees) reported the result of the evaluation by the *Coordenação de Aperfeiçoamento de Pessoal de Nível Superior* (Capes) – grades from 3 to 7 –, however without providing the accreditation ordinance or informing the existence of an accreditation system.

**Figure 3:**
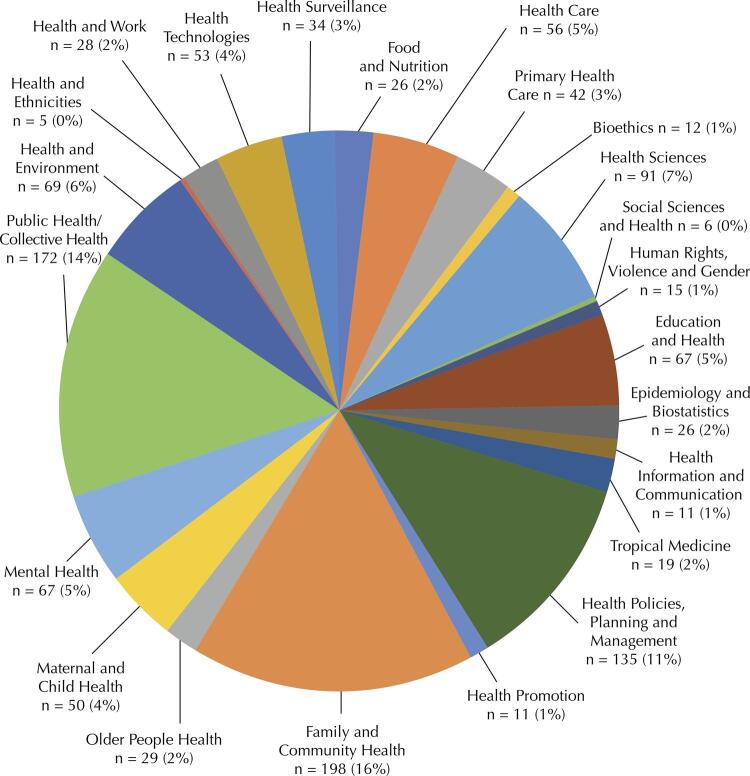
Distribution of public health courses/training programs per area. Brazil, 2022 (n = 1,222).

About the thematic areas in which courses/training programs are distributed within public health field, it was identified that family and community health was the area with the highest number of offers (n = 198; 16%), followed by courses with basic training in public health or collective health (n = 172; 14%) and in the area of health policies, planning, and management (n = 135; 11%) (Figure 3).

**Figure 4:**
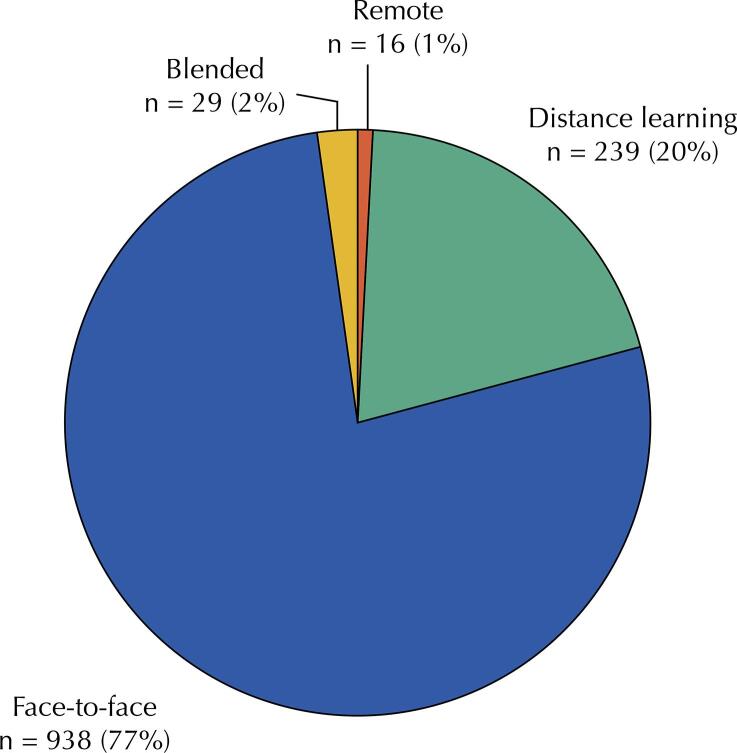
Modality of public health courses and training programs. Brazil, 2022 (n = 1,222).

Regarding the modality adopted by each course/training program, it was identified that 77% of courses and training programs (n = 938) are carried out in person, followed by courses offered in DL (n = 239; 20%) and blended (n = 29; 2%) modalities (Figure 4). As of 2020, due to the covid-19 pandemic and the health measures related to it, courses offered in the “remote learning” modality have been registered, with 100% virtual and synchronous classes (n = 16; 1%).

It is noteworthy that 75% of public health courses and training programs offered in DL (n = 181) modality and 58% of those offered in blended (n = 17) modality are hosted by private institutions. Those data may indicate both public institutions’ preference for offering face-to-face courses/training programs (n = 746; 79%) and private institutions’ greater investment in educational technologies. This study did not have enough data to support either of the two hypotheses, and this issue may be the subject of future investigations.

### Developed Competencies and the Essential Public Health Functions (EPHFs)

The analysis of curricula and study plans of public health courses and training programs offered in the country (Chart) showed that most of them (n = 1,018; 83%) seek to develop professional competencies aligned with the EPHFs, as defined by the WHO in 1998^10^. This original strategy identified nine basic functions associated to public health professionals’ scope of work, which should be developed in training programs. Prevention, surveillance, and control of communicable and non-communicable diseases, monitoring of the health situation, occupational and environmental health and public health management, among other functions, are included in the original nine EPHFs list.

**Chart:** Percentage of courses that consider each of the essential public health functions (EPHF)^10^,^11^, per subject area – Brazil, 2022.

Area	Health policies, planning, and management (%)	Public health (%)	Family and community health (%)
EPHF (WHO, 1998)			
Prevention, surveillance and control of communicable and non-communicable diseases	< 40	< 60	< 60
Monitoring the health situation	< 60	< 60	< 60
Health promotion	< 60	< 60	< 60
Occupational health	< 10	< 40	< 10
Protecting the environment	< 10	< 40	< 10
Public health legislation and regulations	< 60	< 60	< 60
Public health management	< 60	< 60	< 60
Specific public health services	< 60	< 60	< 60
Personal health care for vulnerable and high-risk populations	< 60	< 60	< 60
EPHF (WHO, 2020)			
Monitoring and evaluation of health and well-being, equity, social determinants of health, and health system performance and impact	< 40	< 40	< 40
Public health surveillance; control and management of health risks and emergencies	< 40	< 60	< 60
Promotion and management of health research and knowledge	< 40	< 60	< 60
Development and implementation of health policies and promotion of legislation that protects the health of the population	< 60	< 40	< 40
Social participation and social mobilization, inclusion of strategic actors, and transparency	< 40	< 40	< 60
Development of human resources for health	< 40	< 40	< 40
Ensuring access to and rational use of quality, safe, and effective essential medicines and other health technologies	< 10	< 10	< 10
Efficient and equitable health financing	< 40	< 10	< 10
Equitable access to comprehensive, quality health services	< 40	< 10	< 40
Equitable access to interventions that seek to promote health, reduce risk factors, and promote healthy behaviors	< 40	< 40	< 60
Management and promotion of interventions on the social determinants of health	< 40	< 40	< 40

Nonetheless, when comparing the original EPHFs with the most up-to-date ones – in which these functions were revised and expanded (to 11) in 2020 in an effort coordinated by PAHO/WHO^11^ –, it is observed that only 30% (n = 366) of courses and programs mapped list, in their curricula and study plans, competencies aligned with the updated EPHFs (Chart), and most of which are courses/training programs in family and community health.

According to several authors^2,4,6,9^, the updated EPHFs version^11^contains competencies that are more aligned with the current needs of the public health field of practice in Brazil and Latin America, since they consider the process of social determination of health and the need to face inequalities in health care. In this way, they are closer to the reality that public health professionals will face once their training programs are completed.

Among the three areas of knowledge with the highest number of courses/training programs, the area of health policies, planning, and management had the highest percentage of courses with curricula and study plans aligned with the nine EPHFs, as defined in 1998^10^. The area of family and community health had the highest percentage of courses with study plans aligned with the 11 EPHFs, updated in 2020^11^ (Chart).

Considering all education levels, most of public health courses and training programs identified were offered by public institutions (n = 787; 65% total). However, when it comes to specialization courses, the majority is offered by private institutions. This observation gains special relevance when one identifies, in the analysis of curricula and study plans, that specialization courses have been serving, in the country, as the basic public health training for professionals with different backgrounds. Despite this finding, the data collected and analyzed here do not allow for observing differences, in those curricula and study plans, regrading a greater or lesser alignment between developed competencies and the EPHFs, when comparing courses and programs at the same training level and in the same area of knowledge, offered by public and private institutions. This issue should be the subject of future research.

## DISCUSSION

The analysis of the study results reveals a set of very relevant questions for understanding the role and the scope of public health courses and training programs, which are asymmetrically distributed among the national territory, for health professional development in Brazil.

First, it is necessary to consider the complexity of organizing training processes in a country with continental dimensions and great sociocultural diversity such as Brazil^14,15^. It was evident that there is a greater concentration of public health courses and training programs in regions and states with greater installed training capacity, that is, where training institutions are concentrated.

In a recent study, Santos et al.^8^ point out the unequal distribution of health professionals in Brazil. They highlight that, while the Federal District registers a ratio of 341 physicians per 100,000 inhabitants, in the state of Maranhão this ratio is 82 per 100,000 inhabitants. Regarding the proportion of nurses, the Federal District registers a ratio of 203 per 100,000 inhabitants, while the state of Pará registers 77 per 100,000 inhabitants. Still according to the authors, there are distortions even within the same state, as it is the case of the large number of health professionals and technicians in capitals and metropolitan regions, which concentrate 40% of the Brazilian population, 70% of the physicians and 60% of the nurses working in health services^8^. Such disparities can still be observed within each metropolitan region, and it is difficult to retain health professionals in more vulnerable neighborhoods or areas^8^.

The expansion of public health courses and training programs in Brazil, which began in 1970, was accompanied by a concern, shared by academia and services, about the articulation between curricula and health practices^5^. This concern fostered an intense debate by training institutions on the competencies needed for a more qualified performance in health programs and services^5,16,17^.

According to Garcia^18^, this mobilization also generated a repositioning of spaces for training in public health, since it required the revision of theoretical and methodological references in order to enable the development of skills and competencies aimed at improving the population’s living conditions, through intervention projects conceived in close cooperation with health services and programs.

Nevertheless, despite the undeniable advances observed in the diversity and scope of public health courses and training programs^1,4,5,16,17^, some challenges still remain to overcoming the limited connection between the theoretical contributions of training offers and health professionals’ demands, as identified by different authors^2,5,7,19,20^.

According to Ferla and Rocha^4^, the public health conception present in training programs aimed at health professional is often subjugated by the professional identity nuclei, coined in undergraduate programs in health. Thus, instead of being articulated around an effectively interprofessional practice, they end up overlapping the common field of knowledge by juxtaposition^4^. The authors also draw attention to the role of public health educational institutions in overcoming disciplinary fragmentation and in building spaces for the construction of knowledge that articulate, legitimately, theory and practice to meet the needs of health services and programs users in the country^1,4,5,7,14,17^.

The attempt to overcome disciplinary fragmentation in the training of public health professionals in Brazil narrow the gaps between the developed competencies and skills and the essential public health functions^10,11,14,15,17^. As noted, this connection still needs further strengthening, in order to overcome possible lack of synchrony between the developed competencies and the needs of health professionals who, year after year, complete public health courses and training programs in the country^4,7,9,15 ,7,21,22^.

Another aspect is related to specialization courses, which have been used by health professionals with different basic backgrounds to obtain a basic training in public health. Data from this study reveal that two thirds of the courses at this educational level are offered by private educational institutions. Thus, although it was not possible to observe significant curricular differences between the courses offered by public and private institutions, it is possible to assume the existence of a connection between private offers and market demands, which should be the subject of future studies.

In 2008, Feo^23^ drew attention to the growing participation of private institutions in the training of public health specialists in Latin America, consequently, to a tendency to offer courses to meet specific market needs, instead of health systems’ needs. Particularly considering the weight of private participation in the provision of basic training courses for the Brazilian public health professionals, and the limited articulation between these courses’ study plans/curricula and the EPHFs^10,11^, one could emphasize the need to assess a possible lack of synchrony between professionals’ demands and the competencies developed in public health courses and training programs offered by public and private institutions in Brazil. Moreover, the topic should be inserted in the context of reformulation and improvement of public health training programs in the country, understanding their strategic role for the strengthening of health services, programs, and systems, not only in Brazil, but in Latin America as a whole^1,9,12,4,21^.

## CONCLUSIONS

This study revealed a series of challenges to be overcome by training institutions in Brazil regarding the offer of public health courses and training programs. Those challenges can be understood based on two major gaps: the unequal distribution of public health courses and training programs, which may represent a lack of linkage between installed capacities and the professionals’ training demands; and the limited connection between the competencies developed on these courses and training programs and the EPHFs, as presented in its most current version (2020).

Overcoming the first gap requires structural, political, and technological efforts, facilitating the access of professionals from regions with fewer resources and installed capacities to public health courses and training programs and, thus, promoting the development of skills and competencies among professionals, technicians and other health workers, who will be capable of contributing to the strengthening of health care, surveillance and promotion, locally and regionally. Overcoming the second gap presupposes the disposition, and the necessary conditions, for Brazilian public health training institutions to be able to evaluate their pedagogical practices, improve the competencies they develop in the set of their courses and training programs, and thus, overcome the lack of connection between academic knowledge and professionals’ needs.
