# Supplementary material for: Mapping of public health courses and training programs in Brazil
Source: Rev Saude Publica. 2023 Jul 18;57:45. doi: 10.11606/s1518-8787.2023057005205 (PMC10355314; doi:10.11606/s1518-8787.2023057005205)
Supplement: Translation

# Translation: Mapeamento de cursos e programas de formação em saúde pública oferecidos no Brasil

**Authors:** Frederico Peres, Maria Pasionaria Blanco Centurión, Juliana Monteiro Bastos da Silva, Ana Laura Brandão

**Affiliations:** I Brasil Fundação Oswaldo Cruz. Escola Nacional de Saúde Pública Sergio Arouca. Centro de Estudos da Saúde do Trabalhador e Ecologia Humana. Rio de Janeiro, RJ, Brasil; II Brasil Fundação Oswaldo Cruz. Instituto Nacional de Controle de Qualidade em Saúde. Rio de Janeiro, RJ, Brasil; III Brasil Fundação Oswaldo Cruz. Escola Nacional de Saúde Pública Sergio Arouca. Rio de Janeiro, RJ, Brasil

**Keywords:** Capacitação de Recursos Humanos em Saúde, Educação Profissional em Saúde Pública, Saúde Pública, Brasil

## Abstract

**OBJETIVO:**

Mapear as ofertas formativas em saúde pública no Brasil e identificar, em seus currículos e planos de estudo, as aproximações entre as competências e habilidades neles desenvolvidas e as funções essenciais de saúde pública.

**MÉTODOS:**

Estudo descritivo, exploratório, baseado em levantamento e análise de informações disponíveis nos sites de instituições formadoras que ofertam cursos e programas de formação em saúde pública, no Brasil. Os dados foram levantados no âmbito do *I Mapeo de Cursos y Programas de Formación en Salud Pública de América Latina*.

**RESULTADOS:**

Foram identificados 1.222 cursos e programas de formação em saúde pública oferecidos no país, com distribuição desigual no território, porém com ocorrência em todas as unidades da federação. Os resultados desvelaram um conjunto de desafios à formação de sanitaristas, organizados em torno do distanciamento entre as ofertas e as demandas formativas em saúde pública (distribuição desigual de cursos, concentração de capacidades formativas nas capitais e em determinadas regiões, entre outros) e do distanciamento entre teorias e práticas formativas (baixa vinculação das competências desenvolvidas com as funções essenciais de saúde pública, predomínio da lógica disciplinar orientada por núcleos de competência profissional, entre outros).

**CONCLUSÕES:**

A superação desses desafios requer esforços estruturais, políticos e de apropriação tecnológica, ampliando o acesso dos atores da prática a uma formação vinculada com as demandas dos serviços e programas de saúde no país.

## INTRODUÇÃO

A formação em saúde pública constitui uma ação estratégica para o fortalecimento dos sistemas de saúde e, particularmente, para o desenvolvimento da força de trabalho que atua no planejamento, execução e avaliação de programas, serviços e políticas de saúde^1,2^. Envolve diferentes processos educativos, pelos quais se busca desenvolver habilidades e competências em profissionais/trabalhadores da saúde, em diferentes níveis e com distintos propósitos, articulando saberes acadêmicos com a experiência dos atores da prática, numa perspectiva interdisciplinar e interprofissional^2^.

O Brasil, por sua marcada heterogeneidade sociocultural, dinamismo e desigualdade histórica, tem importância estratégica para pensar e compreender as diferentes dimensões e domínios relacionados à formação em saúde pública^5^. Essa importância é evidenciada sobretudo quando consideramos a incorporação sistemática, nas últimas décadas, de novas formas de pensar e agir sobre os processos saúde-doença-cuidado, incorporando como elementos centrais de análise os determinantes sociais da saúde^3,4,6,7^.

Embora o pensamento sanitarista brasileiro tenha avançado significativamente nas últimas quatro décadas, sua incorporação no conjunto de cursos e programas de formação voltados para o desenvolvimento da força de trabalho em saúde é tímida^4,5^. Isso resulta em um descompasso entre as demandas dos usuários dos serviços e programas de saúde e o escopo das ofertas formativas em saúde pública, distribuídas de forma desigual pelas diferentes regiões do país^5,8^.

Peñaranda-Correa et al.^9^, ao analisarem a formação em saúde pública no Brasil e em outros países da América Latina, apontam a falta de coerência entre teoria e prática, principalmente no que diz respeito à baixa vinculação das práticas pedagógicas e das competências que buscam desenvolver as demandas dos atores da prática e dos usuários dos sistemas de saúde regionais^9^. Como estratégia para superar esse distanciamento, a Organização Mundial da Saúde (OMS) coordenou, na segunda metade da década de 1990, um grupo de trabalho visando identificar um conjunto de funções, no campo da saúde pública, consideradas essenciais para garantir que, independentemente do estágio de desenvolvimento de um país ou região, os sistemas de saúde continuem a responder às necessidades emergentes e prioritárias^10^. Nesse processo foram identificadas nove funções essenciais de saúde pública (Fesp) que, por conseguinte, deveriam orientar as estratégias para o desenvolvimento da força de trabalho em saúde. Em 2020, a Organização Pan-Americana da Saúde (OPS) revisou, atualizou e ampliou para onze Fesp^11^.

Isso posto, e considerando os desafios que se impõem ao desenvolvimento de habilidades e competências interprofissionais na formação de sanitaristas^1,2,6,7,9^, o presente estudo objetiva mapear as ofertas formativas em saúde pública no país e identificar, em seus currículos e planos de estudo, o grau de incorporação das aproximações entre as competências e habilidades neles desenvolvidas e as Fesp^10,11^.

## MÉTODOS

O presente estudo se estruturou a partir do *I Mapeo de Cursos y Programas de Formación en Salud Pública de América Latina*^12^, também elaborado pelos autores deste artigo e cujos dados foram coletados entre novembro de 2021 e abril de 2022, por meio de levantamento sistematizado de informações sobre cursos e programas de formação em saúde pública na América Latina. Nesta pesquisa, foram analisados apenas os dados referentes às ofertas formativas do Brasil, que representaram 53% de todos os cursos e programas de formação identificados no conjunto de países latino-americanos^12^.

O levantamento de dados sobre os cursos e programas de formação, na internet foi realizado com a ferramenta de buscas Google, em português, utilizando inicialmente as palavras-chave “curso”, “programa”, “treinamento”, “formação” e “educação”, sempre associadas aos descritores “saúde pública” ou “saúde coletiva”. Depois, as mesmas palavras-chave foram associadas ao descritor “saúde”, com posterior aplicação dos critérios de exclusão. Por fim, foram utilizados os descritores “universidade” ou “escola de saúde” associados aos descritores “saúde pública”, “saúde coletiva” e “saúde”, para uma busca mais abrangente, a partir dos sites das instituições formadoras. Todos os cursos identificados foram incluídos em uma planilha do Microsoft Excel, para verificação de entradas duplicadas e posterior aplicação dos critérios de exclusão.

Foram excluídos do mapeamento: a) cursos e programas de formação de especialidades médicas, dedicados exclusivamente à prática clínica (mesmo com abordagem interdisciplinar); b) cursos relacionados a especialidades de carreiras específicas da saúde; c) cursos de graduação e de formação técnica; d) cursos autoinstrucionais; e e) os cursos e programas de formação que não possuíam informações disponíveis sobre o currículo ou plano de estudo.

No caso dos cursos técnicos e de graduação em saúde pública/coletiva, a exclusão se deveu ao fato do estudo ter sua origem em um mapeamento regional, abrangendo toda a América Latina, e não somente o Brasil^12^. Esses cursos de graduação representam uma realidade exclusiva do Brasil, o que limita sua compreensão e articulação no contexto regional. Já os cursos técnicos não foram considerados na amostra do presente estudo pelas diferentes concepções que a formação técnica tem na América Latina – há países onde são cursos de nível médio, outros de nível superior, com distintas cargas horárias e duração –, o que igualmente dificulta uma análise regional.

Para cada curso identificado, foram recolhidas as seguintes informações:

- Local: estado e município onde a instituição de formação está localizada e/ou onde a formação é realizada (para cursos e programas com atividades presenciais);
- Nome do curso/programa;
- Nome da instituição;
- Característica da instituição (pública ou privada);
- Nível de formação: cursos e programas de formação contínua, especialização, residência, mestrados e doutorados;
- Modalidade: EaD (cursos estruturados com base em recursos da educação a distância, com momentos síncronos e/ou assíncronos e possibilidade de inclusão de encontros presenciais), presencial, semipresencial (presencial, com atividades a distância, síncronas ou assíncronas) ou de ensino remoto (100% das atividades remotas e síncronas);
- Acreditação ou credenciamento: se o curso possui algum tipo de acreditação ou credenciamento e quem o acredita/credencia;
- Ano da primeira oferta;
- Regularidade da oferta: regular (com frequência anual ou semestral desde a sua primeira oferta) ou eventual (sem frequência definida);
- E dados de contato (site, e-mail, telefone).

Posteriormente, analisaram-se, por meio de técnica de análise de conteúdo descritiva^13^, os programas/currículos das ofertas e os planos de estudos de cada curso, conforme o nível de ensino, identificando em seus tópicos as competências trabalhadas em cada programa ou currículo, bem como as estratégias pedagógicas utilizadas (e.g. aulas expositivas, atividades mediadas por tutores, atividades assíncronas com ou sem mediação etc.), devidamente registradas para fins de descrição das ofertas formativas. Nessa etapa, a análise restringiu-se aos cursos das três áreas temáticas com maior número de ofertas. Para fins de categorização e sistematização, foram consideradas como competências fundamentais aquelas associadas às Fesp^10,11^, e todas as demais consideradas complementares.

Todos os dados (secundários) coletados e analisados no estudo estavam disponíveis, sem restrições, na internet, razão pela qual não se aplicou a análise por comitê de ética em pesquisa.

## RESULTADOS

O levantamento de dados resultou na identificação de 1.222 cursos e programas de formação em saúde pública oferecidos (ou habilitados a serem oferecidos), em todas as unidades da federação (UF) brasileiras. Apesar da grande diversidade de cursos e programas de formação identificados e da ocorrência destes em todas as UF, observou-se maior concentração das ofertas nas capitais e em determinados estados com mais recursos e capacidades instaladas (Figura 1), como Rio de Janeiro e São Paulo, estados com maior número de ofertas registradas.

**Figura 1:** Distribuição das ofertas formativas em saúde pública por unidade federativa. Brasil, 2022 (n = 1.222).

Cabe destacar, porém, o número de cursos ofertados no estado do Amazonas (n = 56), Norte do Brasil, que, apesar de possuir menor capacidade instalada em relação a estados das regiões Sul, Sudeste e Centro-Oeste, apresentou número importante de ofertas e, juntamente com o estado do Pará (n = 42), tem potencial para atender a demandas formativas na região amazônica. Isso inclui também outros países, como Bolívia, Peru, Colômbia e Venezuela, sobretudo considerando que a maior parte dos departamentos (divisões territoriais) fronteiriços desses países não oferecem cursos ou programas de formação em saúde pública^12^ e que a Amazônia tem papel estratégico na garantia da sustentabilidade socioambiental de toda a região.

Quanto ao nível formativo, observou-se um maior número de cursos de especialização (n = 518), correspondendo a 43% de todos os cursos identificados. Em seguida, encontram-se os cursos de mestrado acadêmico (n = 217; 18%) e residência (n = 174; 14%) (Figura 2).

**Figura 2:** Nível das ofertas formativas em saúde pública. Brasil, 2022 (n = 1.222).

É importante observar que 65% dos cursos de especialização identificados são oferecidos por instituições privadas (n = 340). Nos cursos de mestrado (acadêmico e profissional) e doutorado e nas residências a lógica se inverte, com prevalência de ofertas por instituições públicas (89% das ofertas de mestrados acadêmicos; 85% dos mestrados profissionais; 88% dos doutorados; e 83% das residências).

Com relação à regularidade, e considerando apenas os cursos e programas que registravam essa informação (n = 965, ou 79% do total de ofertas), 90% das ofertas formativas eram regulares (periodicidade anual ou bianual), sendo também regulares (anuais) os dois cursos mais antigos registrados, cujas primeiras ofertas remontam ao ano de 1970 (mestrado e doutorado em Saúde Pública da Faculdade de Saúde Pública da Universidade de São Paulo – FSP-USP). Aproximadamente 81% dos cursos e programas de formação foram criados (ou tiveram sua primeira oferta) a partir de 2002.

Já com relação ao credenciamento ou à existência de sistemas de acreditação das ofertas formativas em saúde pública, nenhuma das páginas consultadas registrava tais informações, com exceção do Curso de Residência Multiprofissional em Saúde Materno-Infantil do Hospital de Clínicas de Passo Fundo/RS (informação sobre credenciamento e recredenciamento). Apenas 23% (n = 105) dos programas de pós-graduação *stricto sensu* (mestrados e doutorados) informavam o resultado da avaliação da Coordenação de Aperfeiçoamento de Pessoal de Nível Superior (Capes) – notas de 3 a 7 –, sem, entretanto, registrar a portaria de credenciamento ou existência de sistema de acreditação.

Com relação às áreas temáticas em que se distribuem as ofertas formativas dentro do campo da saúde pública, identificou-se que a saúde da família e da comunidade era a área com maior número de ofertas (n = 198; 16%), seguida dos cursos de formação básica em saúde pública ou saúde coletiva (n = 172; 14%) e da área de políticas, planejamento e gestão (n = 135; 11%) (Figura 3).

**Figura 3:** Distribuição das ofertas formativas em saúde pública por área. Brasil, 2022 (n = 1.222).

Já no que se refere à modalidade adotada por cada oferta formativa, identificou-se que 77% dos cursos e programas de formação (n = 938) são realizados de maneira presencial, seguidos pelos cursos oferecidos na modalidade EaD (n = 239; 20%) e os cursos semipresenciais (n = 29; 2%) (Figura 4). A partir de 2020, por conta da pandemia de covid-19 e das medidas sanitárias a ela relacionadas, observa-se o registro de cursos oferecidos na modalidade “ensino remoto”, com aulas 100% virtuais e síncronas (n = 16; 1%).

**Figura 4:** Modalidade das ofertas formativas em saúde pública. Brasil, 2022 (n = 1.222).

Destaca-se que 75% dos cursos e programas de formação em saúde pública na modalidade EaD (n = 181) são ofertados por instituições privadas, assim como 58% dos cursos na modalidade semipresencial (n = 17). Esses dados podem indicar tanto preferência por ofertas presenciais por parte das instituições públicas (n = 746; 79%) quanto maior investimento em tecnologias educacionais por parte das instituições privadas. O presente estudo não dispôs de dados suficientes para suportar nenhuma das duas hipóteses, podendo essa questão ser objeto de investigações futuras.

### Competências Desenvolvidas e as Funções Essenciais de Saúde Pública

A análise dos currículos e planos de estudo dos cursos e programas de formação em saúde pública oferecidos no país (Quadro), mostrou que a maioria das ofertas formativas (n = 1.018; 83%) busca desenvolver competências profissionais alinhadas às Fesp, tais quais definidas pela OMS em 1998^10^. Essa versão original identificava nove funções básicas para a atuação de um sanitarista, as quais deveriam ser desenvolvidas em ações formativas. Entre as nove Fesp originais incluem-se prevenção, vigilância e controle de doenças transmissíveis e não transmissíveis, acompanhamento da situação de saúde, a saúde ocupacional e ambiental e a gestão em saúde pública, entre outras funções.

**Quadro:** Percentual de cursos que consideram cada uma das funções essenciais da saúde pública^10^,^11^, por área temática – Brasil, 2022.

Área	Políticas, planejamento e gestão em saúde (%)	Saúde pública (%)	Saúde da família e comunidade (%)
Fesp (OMS, 1998)			
Prevenção, vigilância e controle de doenças transmissíveis e não transmissíveis	< 40	> 60	> 60
Acompanhamento da situação de saúde	> 60	> 60	> 60
Promoção de saúde	> 60	> 60	> 60
Saúde ocupacional	< 10	< 40	< 10
Protegendo o meio ambiente	< 10	< 40	< 10
Legislação e regulamentos de saúde pública	> 60	> 60	> 60
Gestão em saúde pública	> 60	> 60	> 60
Serviços específicos de saúde pública	> 60	> 60	> 60
Cuidados pessoais de saúde para populações vulneráveis e de alto risco	> 60	> 60	> 60
Fesp (OPS, 2020)			
Monitoramento e avaliação da saúde e bem-estar, equidade, determinantes sociais da saúde e o desempenho e impacto dos sistemas de saúde	< 40	< 40	< 40
Vigilância em saúde pública, controle e gestão de riscos e emergências em saúde	< 40	> 60	> 60
Promoção e gestão da investigação e do conhecimento na área da saúde	< 40	> 60	> 60
Formulação e implementação de políticas de saúde e promoção de legislação que protege a saúde da população	> 60	< 40	< 40
Participação e mobilização social, inclusão de atores estratégicos e transparência	< 40	< 40	> 60
Desenvolvimento de recursos humanos para a saúde	< 40	< 40	< 40
Garantir o acesso e o uso racional de medicamentos de qualidade, seguros e eficazes e outras tecnologias essenciais de saúde	< 10	< 10	< 10
Financiamento da saúde eficiente e equitativo	< 40	< 10	< 10
Acesso equitativo a serviços de saúde abrangentes e de qualidade	< 40	< 10	< 40
Acesso equitativo a intervenções que visem promover a saúde, reduzir os fatores de risco e promover comportamentos saudáveis	< 40	< 40	> 60
Gestão e promoção de intervenções sobre os determinantes sociais da saúde	< 40	< 40	< 40

Entretanto, quando comparado à versão mais atualizada das Fesp, em que essas funções foram revistas e ampliadas (para 11) em um esforço coordenado pela OPS/OMS em 2020^11^, observa-se que apenas 30% (n = 366) dos cursos e programas de formação mapeados lista, em seus currículos e planos de estudo, competências alinhadas às Fesp atualizadas (Quadro), sendo a maioria ofertas formativas na área da saúde da família e da comunidade.

É importante destacar que a versão atualizada das Fesp^11^ encerra competências que, segundo diversos autores^2,4,6,9^, estão mais alinhadas às necessidades dos campos de prática da saúde pública no Brasil e na América Latina, uma vez que consideram o processo de determinação social da saúde e a necessidade de enfrentamento de desigualdades e da iniquidade na oferta de serviços de saúde. Dessa forma, encontram-se mais próximas à realidade em que os sanitaristas egressos de cursos e programas de formação deverão atuar, uma vez concluídos seus programas formativos.

Dentre as três áreas do conhecimento com maior número de ofertas, a área de políticas, planejamento e gestão em saúde apresentou maior percentual de cursos com currículos e planos de estudos alinhados às nove Fesp, tais quais definidas em 1998^10^. Já a área da saúde da família e da comunidade teve maior percentual de cursos com programas alinhados às 11 Fesp, atualizadas em 2020^11^ (Quadro).

Destaca-se que, por um lado, os cursos e programas de formação em saúde pública identificados são oferecidos em sua maioria por instituições públicas (n = 787; 65% do total), mas, por outro, a maior parte dos cursos de especialização é oferecida por instituições privadas. Tal observação ganha especial relevância quando se identifica, na análise dos currículos e planos de estudo, que os cursos de especialização têm servido à formação básica de sanitaristas, nas suas mais diferentes áreas de atuação. Apesar dessa constatação, os dados aqui levantados e analisados não permitem observar diferenças, nesses currículos e planos de estudo, quanto a um maior ou menor alinhamento entre as competências desenvolvidas e as Fesp, quando comparadas ofertas, no mesmo nível formativo e na mesma área do conhecimento, de instituições públicas e privadas. Essa questão deve ser objeto de investigação futura.

## DISCUSSÃO

A análise dos resultados do presente estudo desvela um conjunto de questões bastante relevantes para a compreensão do papel e da abrangência das ofertas formativas, disponíveis de forma assimétrica no território nacional, voltadas para a formação de sanitaristas.

Primeiramente, é preciso considerar a complexidade de organizar processos formativos em um país com dimensões continentais e grande diversidade sociocultural como o Brasil^14,15^. Ficou evidente que há maior concentração de cursos e programas de formação em saúde pública nas regiões e estados com maior capacidade formativa instalada, ou seja, onde se concentram as instituições formadoras.

Em estudo recente, Santos et al.^8^ apontam a distribuição desigual de profissionais de saúde no Brasil. Destacam que, enquanto o Distrito Federal registra relação de 341 médicos por 100 mil habitantes, no estado do Maranhão essa relação é 82 por 100 mil habitantes. No que se refere à proporção de enfermeiros, o Distrito Federal registra uma relação de 203 por 100 mil habitantes, enquanto o estado do Pará registra 77 por 100 mil habitantes. Ainda segundo os autores, mesmo dentro de um mesmo estado, há distorções, como é o caso da grande concentração de profissionais e técnicos da saúde nas capitais e regiões metropolitanas, que concentram 40% da população brasileira, 70% dos médicos e 60% dos enfermeiros ocupados nos serviços de saúde^8^. Tais disparidades ainda podem ser observadas dentro de cada área metropolitana, havendo maior dificuldade de retenção de profissionais de saúde em bairros ou áreas mais vulnerabilizadas^8^.

A expansão dos cursos de formação em saúde pública no Brasil, iniciada em 1970, veio acompanhada de uma preocupação, compartilhada pela academia e pelos serviços, relacionada à articulação entre os currículos e as práticas de saúde^5^. Tal fato fomentou um intenso debate, pelas instituições formadoras, sobre as competências necessárias para uma atuação mais qualificada nos programas e serviços de saúde^5,16,17^.

Segundo Garcia^18^, essa mobilização também gerou um reposicionamento dos espaços de formação em saúde pública, uma vez que demandou a revisão dos referenciais teóricos e metodológicos de modo a possibilitar o desenvolvimento de habilidades e competências voltadas para a melhoria das condições de vida da população, por meio de projetos de intervenção e da franca cooperação com os serviços e programas de saúde. Entretanto, apesar dos inegáveis avanços observados na diversidade e abrangência das ofertas formativas em saúde pública^1,4,5,16,17^, alguns desafios ainda se apresentam para a superação da limitada vinculação entre os aportes teóricos das ofertas formativas e as demandas dos atores da prática, identificadas por diferentes autores^2,5,7,19,20^.

Segundo Ferla e Rocha^4^, o objeto da saúde pública nos processos de formação de sanitaristas é frequentemente subjugado pelos núcleos de identidade profissional, cunhados nas graduações do campo da saúde. Dessa forma, em vez de se articularem em torno de uma prática efetivamente interprofissional, acabam se sobrepondo ao campo de conhecimentos comum, por justaposição^4^. Os autores chamam, ainda, a atenção para o papel das instituições formadoras em saúde pública na superação da fragmentação disciplinar e na construção de espaços de construção de saberes que articulem, de forma legítima, teoria e prática em prol das necessidades dos usuários dos serviços e programas de saúde no país^1,4,5,7,14,17^.

A tentativa de superar a fragmentação disciplinar na formação de sanitaristas no Brasil passa pela aproximação das competências e habilidades trabalhadas em cursos e programas de formação às funções essenciais da saúde pública^10,11,14,15,17^. Como observado, essa aproximação ainda carece de maior fortalecimento, de forma a superar possíveis dissincronias entre as competências desenvolvidas e as necessidades dos atores da prática que ano após ano egressam dos cursos e programas de formação em saúde pública no país^4,7,9,15,7,21,22^.

Outro aspecto está relacionado aos cursos de especialização, que têm sido utilizados por profissionais graduados em distintas áreas da saúde para obter uma formação básica em saúde pública. Os dados do presente estudo revelam que dois terços dos cursos deste nível formativo são oferecidos por instituições privadas de ensino. Assim, embora não tenha sido possível observar diferenças curriculares significativas entre os cursos ofertados por instituições públicas e privadas, é possível supor a existência de uma vinculação das ofertas privadas às demandas do mercado, o que deve ser objeto de estudos futuros.

Em 2008, Feo^23^ já chamava a atenção para a crescente participação de instituições privadas na formação de especialistas em saúde pública na América Latina e a consequente tendência de oferta de cursos para atender necessidades específicas do mercado, não necessariamente dos sistemas de saúde. Particularmente considerando o peso da participação privada na oferta de cursos de formação básica para os sanitaristas brasileiros e a baixa articulação entre os planos de estudo analisados e as Fesp^10,11^, ressalta-se a necessidade de avaliar as possíveis dissincronias entre as demandas dos atores da prática e as competências trabalhadas nos cursos e programas de formação oferecidos por instituições públicas e privadas no Brasil. Além disso, o tema deve estar inserido no contexto da reformulação e no aprimoramento de cursos de programas de formação em saúde pública, entendendo-os como ações estratégicas para o fortalecimento de serviços, programas e sistemas de saúde, não apenas no Brasil, mas na América Latina como um todo^1,9,12,4,21^.

## CONCLUSÕES

O presente estudo identificou uma série de desafios a serem superados por instituições formadoras em saúde pública no Brasil no que diz respeito à formação de sanitaristas. Esses desafios podem ser compreendidos a partir de dois grandes distanciamentos: a distribuição desigual de cursos e programas de formação em saúde pública, podendo representar um distanciamento entre as capacidades instaladas e as demandas formativas dos atores da prática; e a baixa vinculação entre as competências trabalhadas nos cursos e programas de formação e as Fesp, tais quais apresentadas em sua versão mais atual, de 2020.

A superação do primeiro distanciamento requer esforços estruturais, políticos e de apropriação tecnológica, ampliando o acesso dos atores da prática de regiões com menos recursos e capacidades instaladas a cursos e programas de formação em saúde pública, promovendo o desenvolvimento de habilidades e competências em profissionais, técnicos e outros trabalhadores da saúde, capazes de contribuir para o fortalecimento da atenção, da vigilância e da promoção da saúde, local e regionalmente. Já a superação do segundo distanciamento pressupõe a disposição e as condições necessárias para que as instituições formadoras em saúde pública brasileiras possam avaliar suas práticas pedagógicas, aprimorar as competências que desenvolvem, no conjunto de seus cursos e programas de formação, e assim superar as lacunas entre as teorias que sustentam a formação e as necessidades dos atores da prática.
